# Nanomedicines for Near-Infrared Fluorescent Lifetime-Based Bioimaging

**DOI:** 10.3389/fbioe.2019.00386

**Published:** 2019-12-06

**Authors:** Xianhui Lian, Ming-Yuan Wei, Qiang Ma

**Affiliations:** ^1^Chinese Academy of Inspection and Quarantine, Beijing, China; ^2^School of Life Science and Medicine, Dalian University of Technology, Panjin, China; ^3^Texas Commission on Environmental Quality, Austin, TX, United States

**Keywords:** bioimaging, fluorescence lifetime, near-infrared, nanomaterials, contrast agents

## Abstract

Nanomedicines refer to the application of nanotechnology in disease diagnosis, treatment, and monitoring. Bioimaging provides crucial biological information for disease diagnosis and treatment monitoring. Fluorescent bioimaging shows the advantages of good contrast and a vast variety of signal readouts and yet suffers from imaging depth due to the background noise from the autofluorescence of tissue and light scattering. Near-infrared fluorescent lifetime bioimaging (NIR- FLTB) suppresses such background noises and significantly improves signal-to-background ratio. This article gives an overview of recent advances in NIR- FLTB using organic compounds and nanomaterials as contrast agent (CA). The advantages and disadvantages of each CA are discussed in detail. We survey relevant reports about NIR-FLTB in recent years and summarize important findings or progresses. In addition, emerging hybrid bioimaging techniques are introduced, such as ultrasound-modulated FLTB. The challenges and an outlook for NIR- FLTB development are discussed at the end, aiming to provide references and inspire new ideas for future nanomedicine development.

## Introduction

Nanomedicines refer to the application of nanotechnology in disease diagnosis, treatment, and monitoring (Freitas, 1999). Bioimaging is a technique that uses high-resolution and visualization methods to obtain dynamic changes of the target molecules in cells, in tissues, or *in vivo*. As the signal generator or contrast provider in biological imaging, contrast agents (CAs) have been applied in biomolecular detection, cell imaging, bacterial imaging, cell tracking, vascular imaging, *in vivo* tumor imaging, and treatment, and so on. The application of nanomaterials as CAs of bioimaging is considered one of the nanomedicines for disease diagnosis or monitoring. The success of bioimaging relies heavily on a good CA, but also the progresses of imaging technology.

Various bioimaging techniques are developed to achieve the goal of deep tissue, high resolution, and good contrast. To date, the main bioimaging methods include X-ray imaging, magnetic resonance imaging (MRI), optical bioimaging, electron microscopic imaging, and mass spectrometry imaging. X-ray imaging has larger penetration into tissues than ultraviolet/visible light (Yi Z. et al., 2014; Burdette et al., 2018), but radiation exposure limits its usage and could be a health concern. MRI provides high resolution and great depth for *in vivo* imaging yet suffers from low resolution in the cellular level (Kevadiya et al., 2018; Xu et al., 2018; Wang et al., 2019). Mass spectrometry imaging could be used to investigate the spatial distribution of molecules on complex surfaces (Amstalden van Hove et al., 2010; Bednarczyk et al., 2019; Smith et al., 2019) but not to penetrate tissue. Optical bioimaging provides cellular- or molecular-level information with the advantages of low cost, small size, and noninvasiveness (Kumar et al., 2009; Hui et al., 2010).

Fluorescence bioimaging is one of the earliest appearances in optical bioimaging and has been widely used in biomedical research and clinical stages (Ntziachristos, 2006), thanks to the rapid development of optical technologies and a plethora of emerging fluorescent probes. Fluorescence imaging includes two methods: one imaging targets through a microscope and the other is macroscopic imaging that is based on optical tomography (Pei and Wei, 2019). Good contrast could be obtained for imaging gene, protein, and cellular processes by fluorescence bioimaging (Lv et al., 2015). It has a vast variety of signal readout mechanisms, including fluorescence intensity, lifetime, quenching, or Förster resonance energy transfer (FRET), yet it heavily relies on the function of fluorescent probes. Common fluorescent probe dyes include rhodamines, cyanine dyes, and coumarins. These organic probes may form aggregates that cause aggregation-induced quenching at high concentration, resulting in the decrease of fluorescence intensity (Lu et al., 2012). As for bioimaging in tissue, it faces the challenge that the imaging depth is limited because of the scattering light background.

Near-infrared (NIR) fluorescent probes that are of excitation and emission wavelengths in the 650~900 nm region are attractive for fluorescent bioimaging because interference from the auto-fluorescence background is significantly reduced (Li et al., 2016; Liu et al., 2016). Such bioimaging has been reported for deep-tissue *ex vivo* and *in vivo* imaging (Qian et al., 2009). New NIR fluorescent dyes of rhodamine derivatives have been developed for sensing mitochondrial membrane potential. For instance, Pastierik et al. synthesized and characterized a fluorescent probe, 9–phenylethynylpyronin analogs, conceiving the bathochromic shift to NIR region, which overcame the limits of common xanthene- or rhodamine-based fluorophores (Pastierik et al., 2014). Hang et al. developed a new NIR diketopyrrolopyrrole-based fluorescent dye for protein biosensing and other potential bioimaging (Hang et al., 2014).

Obtaining quantitative information from fluorescent bioimaging is complicated, as the signal is affected by numerous factors, including the excitation light intensity, quenching, and the distribution concentration of fluorophores. Fluorescence lifetime imaging (FLIM) is able to quantitatively obtain the functional information of samples by measuring the fluorescence lifetime of fluorophore, which is independent on the concentration (Becker, 2012). Fluorescence lifetime could be influenced by the microenvironment of the fluorophore, such as temperature, polarity, and the presence of fluorescence quenchers. Because of these advantages, FLIM along with FRET was reported to study the structures, interactions, and functional events between molecules of interest in cells or small animals (Berezin and Achilefu, 2010). Since the lifetimes of autofluorescence in tissue have been reported to be in the range of 0.1–7 ns (Berezin and Achilefu, 2010), the lifetimes of the FLIM CAs would be close to 10 ns or greater to ensure the best contrast or signal-to-background ratio.

The development of a CA that has NIR excitation/emission wavelength and long fluorescence lifetime (>7 ns) appears a key element to the success of deep-tissue high-resolution FLTB. CAs are diverse in nature and common CAs include small molecules and nanomaterials. The latter could be nano-sized (usually <100 nm at one of the dimensions) chemical composites or biomolecules (e.g., fluorescent proteins). In this review, we surveyed the advance in the development of CAs and analyzed their properties for potential use in FLTB, followed by a brief introduction of FLTB instrumentation. Some FLTB results were presented to demonstrate the features and advantages of FLTB. The findings from the development of small molecules and nanomaterial-based CAs are summarized, which will be beneficial for further development of hybrid CAs that small molecule fluorescence dyes are encapsulated in a nano-sized carrier (“dye@nanocarrier”). Finally, a new hybrid bioimaging system that integrates ultrasound with FLTB was described, which may provide a promising platform for future FLTB development.

## NIR Fluorescent Lifetime Bioimaging (NIR-FLTB)

### NIR-FLTB CA

#### Small Molecule-Based CAs

**Figure 1:**
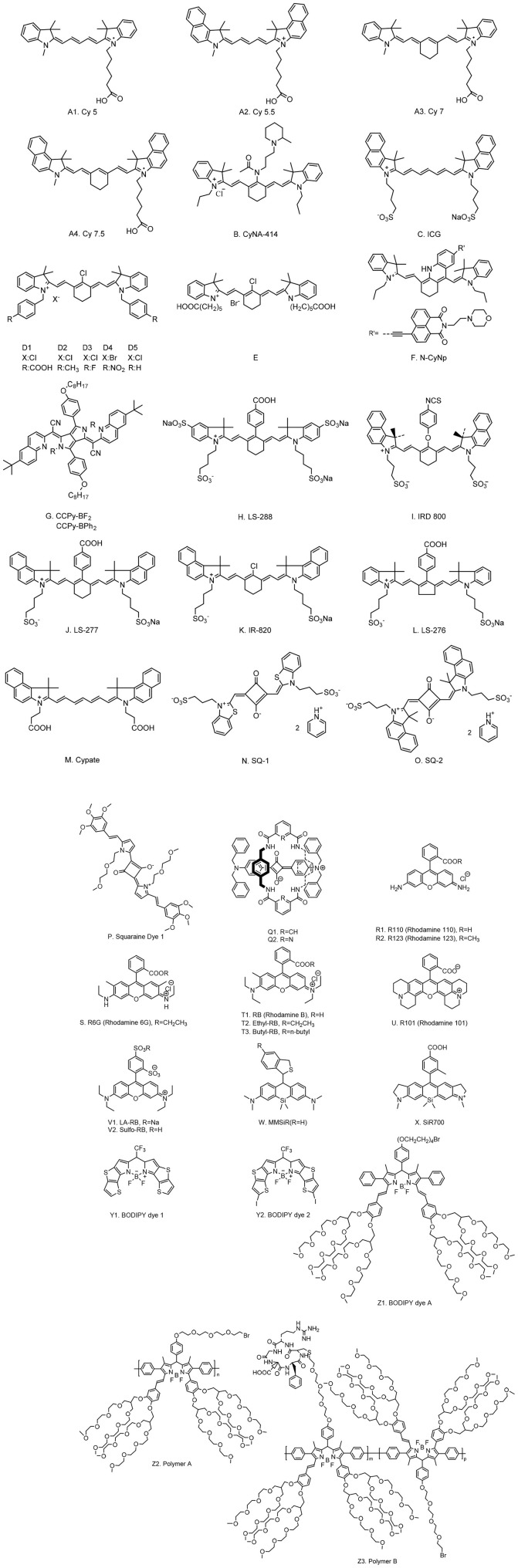
Chemical structures of typical fluorescent lifetime organic dyes.

Serving as CAs, NIR organic dyes have been attracting wide attention in bioimaging applications. Yet only a few of them are directly available because others suffer from poor photostability, hydrophilicity, and stability, as well as low sensitivity in tissues or *in vivo* (Luo et al., 2011; Yi X. et al., 2014). Great efforts have been made to develop new dyes to overcome these issues and to obtain strong florescence intensity and long fluorescent lifetime (Nolting et al., 2011). The most common and frequently studied NIR organic dyes are cyanines, squaraines, phthalocyanines, and porphyrin derivatives, rhodamine analogs, and borondipyrromethane analogs (BODIPYs). The chemical structures of NIR dyes that are of potential to be used for FLTB application are shown in Figure 1, and their photophysical features are summarized in Table 1.

**Table 1:** Photophysical features of typical fluorescent lifetime organic dyes.

**Types of** **organic dyes**	**Organic dyes**	**Solvents**	**Photophysical features**				**References**
			**Absorption** **wavelength** **Abs (nm)**	**Emission** **wavelength** **Em (nm)**	**Lift time** **(*in vitro*)** **(ns)**	**Lift time** **(*in vivo*)** **(ns)**	
Cyanine	Cy 5	H_2_O	651	670	0.98	–	Tinnefeld et al., 2001
	Cy 5.5	–	675	694	–	–	Martinić et al., 2017
	Cy 7	–	755	788	–	–	Martinić et al., 2017
	Cy 7.5	–	788	808	–	–	Martinić et al., 2017
	CyNA-414	–	804	819	–	–	Samanta et al., 2010
	ICG	DMSO	794	831	1.11	0.60–0.84	Berezin et al., 2009a
	D1	Methanol	786	814	–	–	Chen et al., 2006
	D2	Methanol	785	813	–	–	Chen et al., 2006
	D3	Methanol	784	809	–	–	Chen et al., 2006
	D4	Methanol	785	807	–	–	Chen et al., 2006
	D5	Methanol	784	813	–	–	Chen et al., 2006
	E	Methanol	782	808	–	–	Chen et al., 2006
	N-CyNp	HEPES	–	785	–	–	Wu et al., 2017
	pyrrolopyrrole cyanine-BF2 (PPC-BF2)	DMSO	757	779	4.02	3.05–3.80	Berezin et al., 2009a
	pyrrolopyrrole cyanine-BPh2 (PPC-BPh2)	DMSO	824	840	3.35	2.50–2.88	Berezin et al., 2009a
	LS-288	Methanol	770	790	0.81	1.12	Berezin et al., 2009b
	DMSO	Methanol	782	810	0.76	–	Waddell et al., 2000
	LS-277	Methanol	800	811	0.61	0.74	Berezin et al., 2009b
	IR-820	Methanol	820	836	0.25	0.53	Berezin et al., 2009b
	LS-276	Methanol	797	816	0.83	1.12	Berezin et al., 2009b
	Cypate	Methanol	792	817	0.46	0.63	Berezin et al., 2009b
Squaraine	N-propanesulfonate-benzothiazolium squaraine (SQ-1)	Aqueous Solution (BSA)	660 ±1	669 ±1	3.7 ± 0.2	–	Zhang et al., 2013
	N-propanesulfonate-benzoindolium squaraine (SQ-2)	Aqueous Solution (BSA)	675 ± 1	684 ± 1	2.6 ± 0.1	–	Zhang et al., 2013
	Squaraine Dye 1	DMSO	706 ± 1	719 ± 1	0.51 ± 0.07	–	Ahn et al., 2012
	Q1	THF:water (4:1)	650	676	1.24	–	Arunkumar et al., 2005
	Q2	THF:water (4:1)	643	667	6.96	–	Arunkumar et al., 2005
Rhodamine dyes	Rhodamine 110	Water	497	523	4.28	–	Zhang X.-F. et al., 2014
	Rhodamine 123	Water	497	527	4.20	–	Zhang X.-F. et al., 2014
	Rhodamine 6G	Water	525	554	4.22	–	Zhang X.-F. et al., 2014
	Rhodamine B	Water	554	580	1.75	–	Zhang X.-F. et al., 2014
	Ethyl-RB	Water	552	581	1.10	–	Zhang X.-F. et al., 2014
	Butyl-RB	Water	558	584	1.67	–	Zhang X.-F. et al., 2014
	Rhodamine 101	Water	575	604	4.91	–	Zhang X.-F. et al., 2014
	LA-RB	Water	562	588	1.49	–	Zhang X.-F. et al., 2014
	Sulfo-RB	Water	554	591	1.78	–	Zhang X.-F. et al., 2014
BODIPYs	BODIPY dye 1	Hexane	690	693	4.49	–	Sun et al., 2019
	BODIPY dye 2	Hexane	714	716	3.89	–	Sun et al., 2019
	BODIPY dye A	H_2_O	622/665	702	2.6	–	Zhu et al., 2013
	Polymer A	H_2_O	687	711	0.4	–	Zhu et al., 2013
	Polymer B	H_2_O	689	712	0.6	–	Zhu et al., 2013

##### Cyanine dyes

Cyanine dyes were developed first by Williams in 1856 (Williams, 1856). The typical structures were composed of two aromatic nitrogen-containing heterocycle rings linked together by polymethine bridge; see the chemical structure in Figure 1A. The longer the polymethine bridge is (the number in the dye's name represent the number of carbon), the longer the absorption/emission wavelength is (Martinić et al., 2017). For instance, Cy5, Cy5.5, Cy7, and Cy7.5, whose maximum absorption/emission wavelengths (λ_max_/λ_em_) are 652/672, 675/694, 755/788, and 788/808 nm, respectively (Lavis and Raines, 2008; Luo et al., 2011; Martinić et al., 2017). They are widely used in the field of laser materials, paints, and bioimaging for nucleic acids and proteins, and so on; however, it has been noted that they may show poor photostability, undesired self-aggregation, small Stokes shifts, high plasma protein binding rate, and mild fluorescence and low quantum yield in aqueous solution (Levitus and Ranjit, 2011; Luo et al., 2011). There have been great challenges for scientists to improve the performance of cyanine dyes, but some exciting progresses have been witnessed. For example, Samanta et al. (2010) have reported a fluorescent dye, CyNA-414 (see Figure 1B for chemical structure), which has stronger emission intensity and higher photostability than those of indocyanine green (ICG). They claimed that the enhancement was attributed to the introduction of the acetyl group, which is capable of withdrawing electron. Chen et al. (2006) synthesized 3H-indocyanine dyes with different N-substituents (Figures 1D,E), which introduce the electron-donating groups that can obtain better photochemical stability. It was believed that these dyes will be a promising CA in future biological applications. In the process of continuous searching, Stokes shift is also an important factor to be considered in addition to the photochemical stability. In 2017, Wu et al. (2017) developed a NIR cyanine dye, which is modified by naphthalimide with a Stokes shift of c.a. 165 nm and a λ_em_ at 785 nm (Figure 1F). The large Stokes shift mitigates the interference from the excitation light, resulting in a good signal-to-background ratio. It is a promising CA for the imaging of mouse model according to their results. Unfortunately, the fluorescence lifetimes of these modified cyanine dyes have not been studied; however, the findings from these reports will offer a meaningful strategy for future lifetime studies.

The Food and Drug Administration (FDA) of the United States approved ICG dye as the only cyanine probe for *in vivo* use in biomedical applications. However, ICG has been rarely used in fluorescence lifetime bioimaging due to its short lifetime (<2 ns). As for other cyanine dyes, increasing efforts have been taken to improve the fluorescence lifetime to meet the requirements of FLTB. It appears that none of these dyes shows a fluorescent lifetime being close to 7 ns (the background noise of the maximum fluorescence lifetime from tissue). Due to the lack of the fluorescence lifetime data of the abovementioned modified cyanine dyes, we cannot put this as a dead-end for FLTB yet.

##### Squaraine dyes

Squaraine dyes show intensive absorption/emission in the visible and NIR regions. These compounds belong to the subclass of polymethoxy dyes, which consist of an oxocyclobutenolate core with aromatic or heterocylic components at both ends of these molecules (Patsenker et al., 2011). Squaraine dyes have been widely used in the fields of printing and dyeing, photo-detector, biological probe, photodynamic therapy, optical data storage, laser printing, optical-emitting effect transistor, non-linear optics, infrared photography, and solar cells (Hu et al., 2013) because of the advantages of intensive absorption bands and good photoconductivity; however, they show small Stokes shifts and poor solubility in aqueous solution (resulting in aggregate and quenching). To this end, Arunkumar et al. (2006) encapsulated dyes in the amide-containing macrocycle, and they claimed that the method could be extended to other dyes. It is believed that the fluorescence properties and the chemical robustness of squaraine dyes can be adjusted by modifying or adding moieties into the dye. A few squaraine dyes and their photophysical properties were summarized in Table 1.

##### Rhodamine dyes

**Figure 2:**
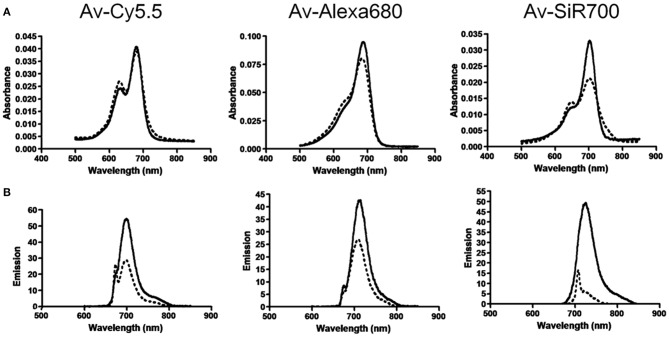
**(A)** Absorbance spectra of near-infrared (NIR) imaging probes, consisting of a NIR fluorophore conjugated to avidin, without SDS (dashed line) and with SDS (solid line). **(B)** Emission spectra of NIR imaging probes without (dashed line) and with (solid line) SDS (McCann et al., 2011). Reproduced with permission. Copyright 2011 American Chemical Society.

Rhodamine dyes belong to the class of xanthene and have been extensively used as fluorescent probes due to their distinguishing photophysical properties, such as water solubility (Guo et al., 2014). The chemical structures of some rhodamine dyes/rhodamine analogs are shown in Figure 1. Although they have prodigious molar extinction coefficients and resistance, their emission wavelengths are rarely above 600 nm. For instance, rhodamine B, rhodamine 6G, and rhodamine 101 and their emission wavelengths are all in the visible region (~600 nm) (Prazeres et al., 2008; Berezin and Achilefu, 2010). This limits the application of rhodamine dyes in *in vivo* bioimaging. In recent years, developing new rhodamine analogs by modifying their xanthene core has received great attention. Koide et al. (2011) reported a far-red to NIR fluorescence probe, MMSiR, which was designed based on Si-rhodamine. Its fluorescence shows no dependence on pH and high resistance to autoxidation and photobleaching. Moreover, it had been used for real-time imaging. McCann et al. (2011) compared a silica-rhodamine-based NIR fluorophore (SiR700) with cyanine-based dyes. The absorbance (2A) and emission (2B) spectra of the dye-conjugated avidin are represented in Figure 2. In the presence of a surfactant, sodium dodecyl sulfate (SDS), the emission intensity of the dye-conjugated avidin was enhanced by c.a. 3-folds. This indicates that the decreased polarity (more hydrophobic) generated from the formation of SDS micelle could lead to the enhancement of fluorescence emission intensity. Their findings suggest that SiR700 could be useful for *in vivo* optical imaging.

##### BODIPY dyes

BODIPY dyes have a corporate structure of 4,4′-difluoro-4-bora-3a,4a-diaza-s-indacene, which were discovered by Treibs and Kreuzer in 1968 (Treibs and Kreuzer, 1968). These dyes are of high extinction coefficient, strong fluorescence intensity, good photostability, and inertness to pH and medium (Geddes and Lakowicz, 2005). Normally, BODIPYs dyes' fluorescence emission wavelengths are close to NIR range. Adjusting the absorption/emission wavelengths of these dyes to the NIR range can be realized by modifying the core of dyes. For instance, Zhu et al. (2013) prepared a polymeric BODIPY dye bearing arginine–glycine–aspartic acid (RGD) peptides (polymer B). The thiol-functionalized RGD cancer-homing peptide was conjugated with tetra(ethylene glycol) tethered spacers (polymer A) under a mild basic condition. The water-soluble BODIPY dye was used for NIR fluorescence imaging of breast cancer cells. Sun et al. (2019) designed two thieno[3,2-b]thiophene-fused BODIPY derivatives, which have an enhanced absorption in the NIR region. Study showed that BODIPY dyes are highly sensitive to microenvironment (Pei and Wei, 2019), yielding to a fact that their fluorescence intensity and lifetime dropped significantly when transferred from organic solvent to water phase. This hinders scientists using them directly in FLTB applications.

##### Summary of small molecule-based CAs

We summarized the chemical structures, absorbance/emission wavelengths, and fluorescence lifetime (solvents) of some representative NIR organic dyes, including cyanine, squaraine, phthalocyanines and porphyrin derivatives, rhodamine analogs, and BODIPY analogs. New dyes had been developed to improve either water solubility or fluorescence properties. These findings encourage scientists to conduct in-depth studies, one of which might be the improvement of their fluorescence lifetimes. As for NIR FLTB application, the fluorescence lifetime threshold from tissue background noise was found to be up to 7 ns. None of these dyes can be perfectly qualified to use as a CA for FLTB in tissue. As seen in Table 1, some BODIPY dyes show promising lifetimes, for example, ~4 ns, in organic solvent, but their lifetimes dropped significantly in aqueous solution or buffer solutions. A trade-off between water solubility and fluorescence lifetime often exists. To this end, dye-encapsulated nanomaterials (“dyes@nanocarrier”) might be a solution. The hydrophobic internal nanocore offers an environment to maintain the long lifetime of the dyes, whereas the external hydrophilic nano-shell improves the water solubility. Such composites were potentially meeting the requirements of FLTB CAs. An outstanding example is to encapsulate BODIPY dyes into a micelle (Pei and Wei, 2019). The resulting micelle-encapsulated dye shows great water solubility but also the unchanged fluorescent lifetime of the BODIPY dye.

#### Nanomaterial-Based CAs

Nanomaterials are a class of materials that are of size range from 1 to 100 nm for at least one dimension. Compared with molecular probes, nanomaterial-based fluorescence probes are not subject to nonspecific binding of proteins, so their optical properties are not affected. In recent years, the research of nanomaterial-based CAs has been developing rapidly, especially in the fields of medicine and diagnosis (Hahn et al., 2011). Their fluorescence appeared not be easily affected by solvent polarity, ionic strength, pH, and temperature. More importantly, they show high sensitivity and selectivity to the target and have good contrast (Wolfbeis, 2015). The most commonly used nanomaterial-based CAs are hydrophobic and hydrophilic organic polymers, nanoparticles made of silica and organically modified silica, quantum dots (QDs), semiconducting organic polymers, carbonaceous nanomaterials including carbon (quantum) dots, carbon nanoclusters and carbon nanotubes, nanodiamonds, upconversion materials, metal particles, metal oxides (Wolfbeis, 2015). The photophysical features of selected nanomaterials are shown in Table 2. As discussed earlier, to eliminate the autofluorescence from tissue, the fluorescence lifetimes for FLTB CAs are recommended to be equal or above 10 ns. As shown in Table 2, QDs, nanodiamonds, gold clusters, and upconverting nanoparticles (UCNPs) are qualified for time-gated FLTB. Among them, QDs and UCNPs have unique advantages, such as NIR emission, long fluorescence lifetime, and tunable fluorescence properties, which make them promising CAs for NIR-FLTB. The detailed features and applications of QDs and UCNPs are described below. Gold cluster- and nanodiamond-based CAs are briefly introduced because of their relatively shorter excitation wavelengths (Table 2).

**Table 2:** Photophysical features of selected nanomaterials^§^.

	**Excitation range [nm]**	**ε [M^**−1**^cm^**−1**^]**	**Γ [%]**	**Fluorescence lifetime**	**Time-gating**	**Blinking**	**Photostability**
Quantum dots	700–1,300	10^5^-10^6^	10–90	20 ns−5 μs	Yes	Yes	High
Polymer dots	500–800	10^6^-10^7^	10–50	≤1 ns	No	No	Medium
Nanodiamonds (NV)^*^	520–580	10^6^	70–80	10–30 ns	Yes^*^	No	Very high
Organic dyes	600–800	10^4^-10^5^	10–50	<1–6 ns	No	Yes	Low
Carbon dots	500–650	10^4^-10^5^	1–10	≤10 ns	No	No	Medium
Gold clusters	500–650	10^4^-10^5^	<1–3	3–800 ns	Yes	Yes	Medium
Carbon nanotubes^**^	700–1,300	10^7^	<<1–7	≤1 ns	No	No	High
Graphene oxide	400–650	10^4^	<1–5	≤1 ns	No	Yes	Medium
UCNPs	980	10^3^-10^4^	<1–7	>100 μs	Yes	No	High

§ *Adapted from Reineck and Gibson (2016) with permission. Copyrights 2017 John Wiley. ε, the molar absorption coefficient; Γ, the fluorescence quantum yield; UCNPs, upconversion nanoparticles*.

* *Many nanodiamonds's lifetimes are not long enough for time-gating, except for the one that is of >60 nm in diameter (Hui et al., 2014)*.

** *Absorption coefficient is proportional to nanotube length. The value is based on a length of 200 nm*.

##### Quantum dots

**Figure 3:**
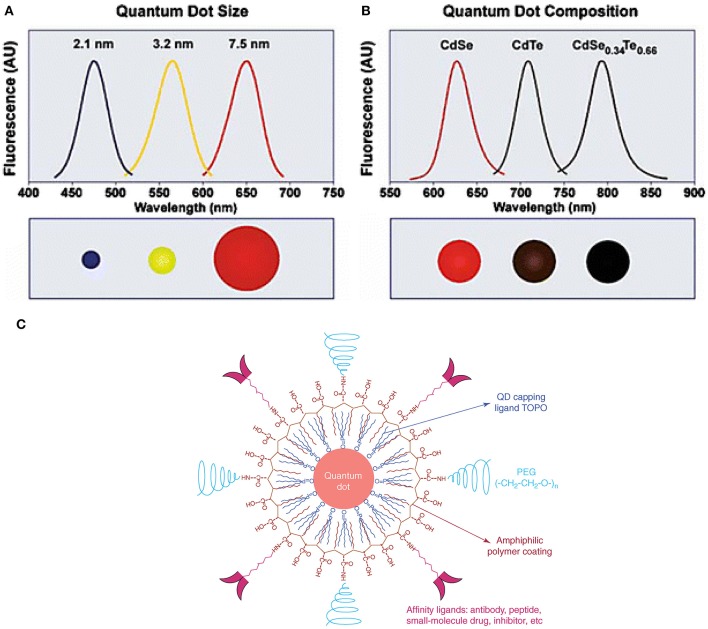
QDs structure and novel optical properties: **(A)** Size and composition tuning of optical emission for binary CdSe and ternary CdSeTe quantum dots. A CdSe QD with various sizes (given as diameter) may be tuned to emit throughout the visible region by changing the nanoparticle size while keeping the composition constant. **(B)** The size of QD may also be held constant, and the composition may be used to alter the emission wavelength. In the above example, 5-nm-diameter quantum dots of the ternary alloy CdSe_x_Te_1−x_ may be tuned to emit at longer wavelengths than either of the binary compounds CdSe and CdTe because of a non-linear relationship between the alloy bandgap energy and composition (the spectrum maximum near 790 nm corresponds to CdSe_0.34_Te_0.66_). **(C)** The structure of a multifunctional QD probe. Schematic illustration showing the capping ligand TOPO, an encapsulating copolymer layer, tumor-targeting ligands (such as peptides, antibodies or small-molecule inhibitors), and polyethylene glycol (PEG) (Smith et al., 2004; Gao et al., 2005). Reproduced with permission. Copyrights 2004 John Wiley and Sons and 2005 Elsevier.

Quantum dots (QDs), tiny light-emitting particles at nanometer-size, are extremely bright and photostable. Although QDs can be excited by a wide range of wavelengths, the NIR QDs are considered safe because they are non-ionizing. QDs are emerging as a new class of fluorescent CAs for bioimaging, thanks to the high sensitivity (brightness), high specificity (targeting), and the capability for multiple targets (narrow emission spectrum). A number of NIR fluorescent QDs have been designed, including HgS, PbS, PbSe, Ag_2_S, and so on. HgS can exhibit narrow, size-dependent transitions between 500 and 800 nm for sizes ranging from 1 to 5 nm (Goswami et al., 2012); PbS has a large exciton Bohr radii of 20 nm, whose fluorescence has a wider adjustable range (Wang et al., 2012); PbSe belongs to e IV-VI semiconductors, which has large Bohr radius, small Stokes shift, and bright luminescence (Tan et al., 2007); Ag_2_S belongs to I-VI semiconductors, which has 1.0 eV of narrow band gap and low toxicity (Meherzi-Maghraoui et al., 1996). The emission wavelength of a QD is related to size, which can be adjusted through changing surface composition (Bentolila et al., 2009). For example, emission wavelength of cadmium selenide (CdSe) QDs can be tuned to cover 450–650 nm range by adjusting the size of QD from 2 to 7 nm (Figure 3A). By changing the composition of the alloy CdSe_x_Te_1−x_, emission wavelength of QD with a diameter of 5 nm can be tuned from 610 to 800 nm (Figure 3B) (Smith et al., 2004). Compared with organic molecule-based CAs, QDs show more intriguing fluorescence properties, including adjustable emission wavelengths, high molar extinction coefficient, high fluorescence quantum yield, large effective Stokes shift, and high photobleaching resistance (Hahn et al., 2011; Zhang et al., 2012). According to the survey, the core of the most commonly used QDs is CdSe coupled with the shell layer of ZnSe or CdSe. For bioimaging applications, the core-shell structure is often coated with surfactants (e.g., stabilizer ligands, amphiphilic polymers) or affinity ligands (e.g., antibodies), as shown in Figure 3C (Xing and Rao, 2008). Most importantly, QDs have a long fluorescence lifetime, ranging from 20 to 50 ns or greater, which overcame the limit of organic molecules in FLTB. The relatively long fluorescence lifetime separates QDs' fluorescence from fluorescence background from tissue (<7 ns) (Smith et al., 2004). Although QDs with emission wavelengths of 450–650 nm have been well-studied, QDs that emit at NIR wavelengths are rarely explored because of the challenging synthesis (He et al., 2011).

**Figure 4:**
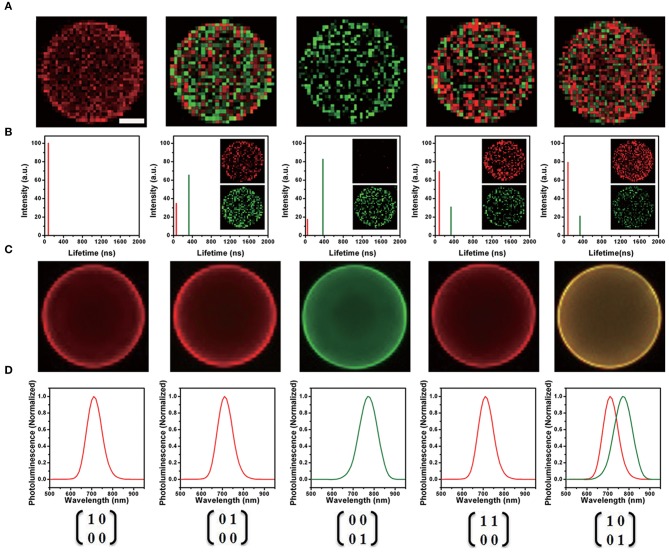
**(A)** Fluorescence lifetime imaging (FLIM) image of each near-infrared (NIR)-emitting 2D encoded microbeads. The scale bar indicates 10 μm, which is also applicable for **(C)**. **(B)** Lifetime distribution of NIR-emitting 2D encoded microbeads (inset: unmixed FLIM image). **(C)** Merged fluorescence emission spectra image of each NIR emitting 2D encoded microbeads with multi-emission (pseudo-colored red: 700 nm; pseudo-colored green: 760 nm). **(D)** Fluorescence emission spectra of NIR-emitting 2D encoded microbeads (Chen et al., 2014). Reproduced with permission. Copyright 2014 John Wiley and Sons.

In 2014, Chen and his collaborators (Chen et al., 2014) reported a new class of lattice-strained CdTe/CdS: Cu QDs, which have high photoluminescence quantum yield (PL QY) (50–70%), widely tunable NIR-fluorescence emission spectrum (700–910 nm), and long fluorescence lifetime (up to around 1 μs). They used the as-prepared QDs to fabricate the NIR-emitting 2D codes based on multi-emission and multi-lifetime. The FLIM images and lifetime distribution of microbeads are presented in Figure 4.

**Figure 5:**
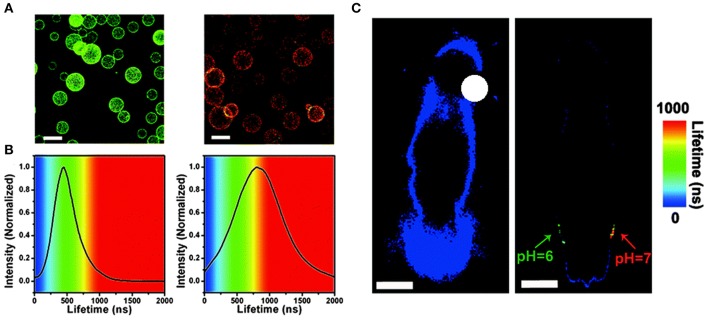
**(A)** Fluorescence lifetime imaging (FLIM) images of microbeads equipped with QDs-720, dispersed in buffers with different pH values (left: pH = 6.0, right: pH = 7.0, scale bar: 100 mm) and **(B)** PL lifetime histograms collected from the images. **(C)** *In vivo* FLIM experiments of the background of the nude mouse (left) and the QDs-720 injected into adjacent locations with different pH values (green: pH = 6.0, red: pH = 7.0) on the back of the nude mouse (right), respectively (scale bar: 10 mm) (Chen et al., 2015). Reproduced with permission. Copyright 2015 Royal Society of Chemistry.

In 2015, Chen et al. prepared Cu-doped CdZnS QDs that have ultra-small size (~3.5 nm), NIR-emission (~720 nm), and long lifetime (up to ~1 μs) (Chen et al., 2015). The QDs-based *in vivo* pH imaging using FLIM can be seen in Figure 5. Autofluorescence from the tissue was suppressed in Figure 5C, and the CAs showed a great selectivity toward pH. In 2019, Pons and his colleagues reported that the fluorescence lifetimes of ZnCuInSe/ZnS QDs are between 100 and 300 ns, which enable the efficient rejection of fast autofluorescence photons but also increase the sensitivity (Pons et al., 2019). With the excitement of conceiving FLTB with long fluorescence lifetime QDs, scientists also noted that such organic QDs may have toxicity *in vivo*, such as genotoxicity (Giraud et al., 2009).

##### UCNPs

**Figure 6:**
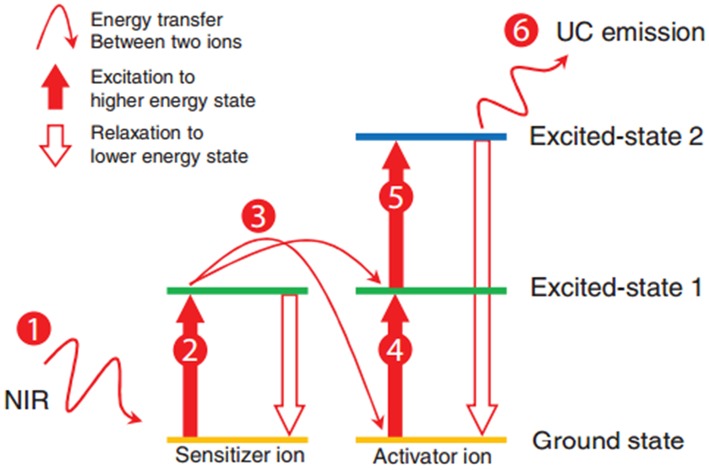
Schematic of the energy transfer upconversion mechanism (Tan et al., 2016). Reproduced with permission. Copyright 2016 John Wiley and Sons.

UCNPs were first described in the early twentieth century, and UCNPs convert long-wave light into shorter-wave luminescence and have long fluorescence lifetime (Auzel, 2004). UCNPs can emit photons with higher energy than the absorbed photons. UCNPs have high photostability, deep tissue penetration (excited at NIR wavelengths), and low background interference (Liu et al., 2014), by which it attracts increasing attention in the field of bioimaging. The mechanism of energy transfer upconversion is describe in Figure 6 (Tan et al., 2016). UCNPs are often excited at NIR wavelengths (far NIR excitation wavelength will eliminate the auto-fluorescence background), and the resulting emission occurs in visible or ultraviolet regions of the electromagnetic spectrum.

**Figure 7:**
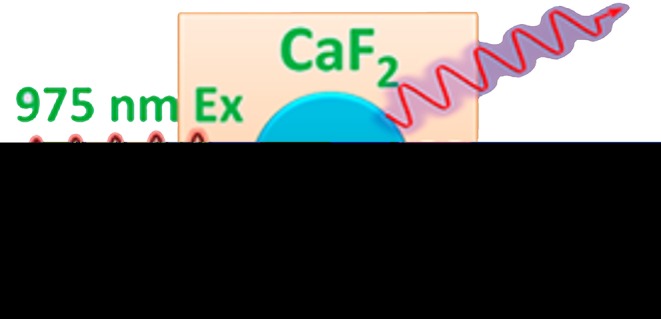
Structure of (α-NaYbF4:Tm^3+^)/CaF_2_ upconverting nanoparticles (Chen et al., 2012). Reproduced with permission. Copyright 2012 American Chemical Society.

At the early stage of the development of UCNPs, efforts had been made to improve the upconversion efficiency by modifying composition, phase, and size, whereas the quantum yield of UCNPs was still limited by energy transfer between doped ions. The most exciting feature of UCNPs is that their fluorescence lifetime reaches microseconds range (Kim and Kang, 2010). In 2009, Hilderbrand et al. reported a multi-channel luminescent Y_2_O_3_-based UCNP, and it was used for the *in vivo* imaging of blood vessels (Hilderbrand et al., 2009). Thanks to the long fluorescence lifetime of the UCNPs, the good contrast images were obtained after a microsecond exposure, which eliminated the background fluorescence from the tissue. Wu et al. reported lanthanide-doped UCNPs that are of NaYF_4_ (β-NaYF_4_) nanocrystals with multiple Yb^3+^ and Er^3+^ dopants, and the UCNPs showed no on/off emission behavior (“blinking”) down to milliseconds (Wu et al., 2009). *In vivo* and *in vitro* bioimaging results demonstrated the capability of such UCNPs toward single molecular bioimaging and future targeting bioimaging applications. Chen *et al*. described a high quantum yield UCNPs with core/shell structure of (α-NaYbF_4_:Tm^3+^)/CaF_2_ (Figure 7) (Chen et al., 2012). It exhibits highly efficient NIR_in_-NIR_out_ upconversion, and it was used for high contrast and deep bioimaging.

##### Gold clusters and fluorescent nano-diamonds

Gold clusters often consist of several tens of gold atoms, prepared through the chemical reduction of gold ions in solution or the etching of large gold NPs. Their fluorescence emissions could be tuned into NIR range (usually up to ~700 nm) with lifetime up to ~800 ns (Reineck and Gibson, 2016). Thanks to the long fluorescence lifetime, they have been used for bioimaging applications (Shang et al., 2011; Liu et al., 2013; Roy et al., 2015), see Table 2. Likewise, the fluorescence lifetime of nano-diamonds could be up to ~20 ns (Hui et al., 2014), which makes them qualified for bioimaging application. For both cases, researchers employed time-gated fluorescence to observe fluorescence intensity after applying a 10-ns threshold.

##### Fluorescent proteins

The synthesis of fluorescent proteins includes two processes: the first is encoding by a cascade of DNA, including gene transcription and translation; and the second is maturation and form fluorophore (Berezin and Achilefu, 2010). The fluorophores are protected by the protein shell, which can isolate fluorophores from the external environment, and thus their lifetimes are stable and not sensitive to external environment. However, their optical properties could be affected by pH that may cause the conformation change of the protein. A cerulean (ECFP/S72A/Y145A/H148D) fluorescent protein was reported that possesses high extinction coefficient and a fluorescence lifetime of 3.5 ns (Rizzo et al., 2004).

##### Summary of nanomaterial-based CAs

As shown in Table 3, both QDs and UCNPs show acceptable fluorescence lifetime (>10 ns) at NIR region, which meet the criteria of FLTB CAs. The instinct features of QDs fluorescence are the narrow emission spectrum and adjustable emission wavelength. These advantages make them promising CAs candidates for multiple targets bioimaging. Using UCNPs as CAs allows the ease of low power excitation light source and of the elimination of auto-fluorescence background. The excitation light for UCNPs could be at far NIR, such as 950 nm, under which rare fluorescence from tissue could be generated. Not to mention that their long fluorescence lifetime offers a great tool to further improve the signal-to-background ratio (SBR). The SBR of UCNPs-based bioimaging was adequate for fluorescence intensity-based bioimaging; thus, few studies have investigated UCNPs-based FLTB. Biocompatible surfactants, such as PEG, could be coated onto the surface of QDs and UCNPs, which helps in mitigating the toxicity of such CAs during *in vivo* bioimaging. However, increasing concerns have been brought up by toxicologists regarding the toxicity of the core components of QDs and UCNPs. Fortunately, fluorescence protein might be a nontoxic alternative to QDs or UCNPs. Yet the fluorescence lifetimes of fluorescence proteins fall short at a few nanoseconds, which is not beneficial to outstand the autofluorescence background from tissue (up to ~7 ns). Even though good contrasts have been conceived, light scattering in tissue is still a challenge for nanomaterial-based FLTB to improve the resolution in deep tissue.

**Table 3:** Photophysical features of typical nanomaterials-based contrast agents (CAs).

**Type**	**Fluorescent** **component**	**Solvents**	**Photophysical features**				**References**
			**Absorption** **wavelength** **Abs (nm)**	**Emission wavelength** **Em (nm)**	**Lift time** **(*in vitro*)** **(ns)**	**Lift time** **(*in vivo*)** **(ns)**	
Quantum Dots (QDs)	HgS	H_2_O	550	730	~5.2	–	Goswami et al., 2012
	PbS	toluene	770	960	2,570	–	Cheng et al., 2017
		hexane	770	960	2,820	–	
		chloroform	770	960	2,710	–	
	PbSe	H_2_O	–	–	8,670	–	Kigel et al., 2009
	Ag_2_S	Chloroform	785	975–1175	57–181	–	Zhang Y. et al., 2014
		water	808	1200	~50	–	Santos et al., 2017
	CdSe	H_2_O	425	640	1.68	–	Zhang et al., 2019
	CdSe@ZnS	PBS	–	605	~43	–	Gaigalas et al., 2014
		PBS	–	705	131	–	
		H_2_O	–	800	160	–	
	CdTe/CdS: Cu	water	–	700–910	1,000	–	Chen et al., 2014
	Cu-doped CdZnS	Buffer	–	720	~800 (pH 7)	~800 (pH7)	Chen et al., 2015
	ZnCuInSe@ZnS	blood stream	–	~830	–	100–300	Pons et al., 2019
Upconversion nanoparticles (UCNPs)	Y_2_O_3_-based	Blood stream	980	795	–	~ms	Hilderbrand et al., 2009
	NaYF_4_ (β-NaYF_4_) nanocrystals with multiple Yb^3+^ and Er^3+^ dopants	Dulbecco's Modified Eagle Medium (DMEM)	980	540 & 650	~ms	~ms	Wu et al., 2009
	(α-NaYbF_4_:Tm^3+^)@CaF_2_	water	975	800	3 × 10^5^	>10^5^ (tissue)	Chen et al., 2012
Gold Clusters	Au_22_	water	561	628	245.1	–	Roy et al., 2015
	Au/dihydroplipoic acid	PBS	550	684	500–800	>600 (cell)	Shang et al., 2011
	Au/trysine	water	520	700	~1,000	–	Liu et al., 2013
Nanodimanonds	Fluorescent nanodiamonds	water	532	700	~20	–	Hui et al., 2014

#### FLTB Instrumentation

**Figure 8:**
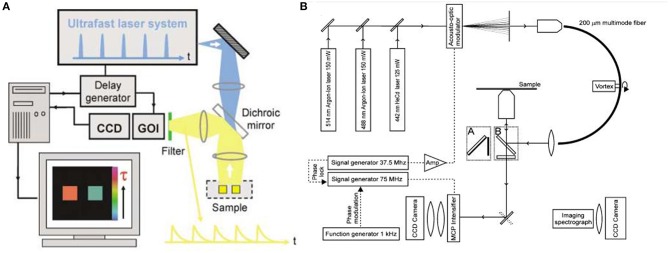
**(A)** Schematic of wide-field time-domain fluorescence lifetime imaging (FLIM). **(B)** Setup of the frequency-domain FLIM instrumentation. (Elson et al., 2004; Van Munster and Gadella, 2004). Reproduced with permission. Copyrights 2004 Royal Society of Chemistry and 2004 John Wiley and Sons.

FLIM mainly includes time- and frequency-domain methods (Birch and Hungerford, 2002). Their main components can be seen in Figure 8. The spectroscopic configuration is mainly composed of excitation source and detection instrumentation (Leblond et al., 2010). For time-domain method, fluorophores were excited by pulsed light sources, and fluorescence lifetime measurement is conducted with time-gated detection, time correlated single photon counting (TCSPC), or streak-FLIM. Microchannel plate and intensified charge-coupled device (CCD) camera can realize full-field FLIM (Wang et al., 1992); multi-photon excitation FLIM can be performed by combination confocal microscopy or multiphoton excitation fluorescence microscopy with TCSPC (Becker, 2012); streak-FLIM can be achieved by a streak camera (Krishnan et al., 2003). As for frequency-domain method, the typical light sources are light-emitting diodes or laser diodes. Unlike time domain, it uses a co-frequency modulated CCD or photomultiplier tube (PMT) as a detector that demodulates and receives fluorescent signals. When FLIM instrument is applied into biological samples, an inevitable issue is the scattering light in deep tissue. For instance, the imaging depth for breast could reach centimeters. In such scenarios, the resolution of optical imaging technique will be lost.

#### Novel Hybrid FLTB Technology

**Figure 9:**
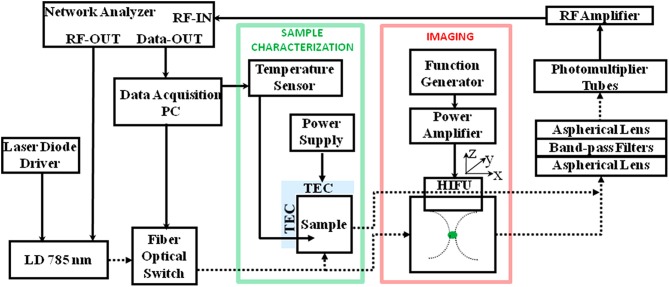
Diagram of the frequency domain ultrasound-switchable fluorescence (USF) system. The same instrumentation is used both for characterizing the sample agent and for imaging experiments. The sample characterization setup is indicated by the green box, whereas the imaging setup is indicated by the red box (Lin et al., 2012). Reproduced with permission. Copyright 2012 Society of Photo-Optical Instrumentation Engineers (SPIE).

Ultrasound-based bioimaging is well-known for its great penetration in tissue and good resolution; however, it does not have the good contrast and vast detection method as fluorescence bioimaging. Theoretically, the combination of ultrasound and fluorescence could harness advantages of both and conceive deep-tissue, high-resolution, and good contrast bioimaging. Ultrasound-switchable fluorescence (USF) is a technology that uses ultrasound pulses to make fluorophores emit fluoresce in the ultrasound focus region. In 2012, USF bioimaging was first reported, and the method is about one order of magnitude better than other deep-tissue fluorescence imaging (Yuan et al., 2012). Another research group demonstrated a frequency-domain temperature-modulated fluorescence tomography system (Lin et al., 2012). This work realized a depth of 2 cm and a size of 3 mm in the biological tissue. The measurement system and experimental setup are shown in Figure 9.

It is noteworthy that USF CAs are a hybrid CA of small molecule and nanomaterial. NIR dyes, that is, ICG or BODIPY, were encapsulated in a polymer nano-carrier, for example, Poly(N-isopropylacrylamide) (PNIPAM) nanoparticles or Pluronic micelles. The dyes' fluorescence is highly dependent on the polarity of the microenvironment; that is, higher intensity of emission at more hydrophobic environment. The microenvironment inside the nano-carrier will become more hydrophobic through conformation change upon being heated, namely, thermosensitive property. When the ultrasound (high-intensity focused ultrasound, HIFU) gave the energy (heat) at the focused area, the USF CAs within the area will emit higher fluorescence (turned ON). Intensity-based USF bioimaging has been explored (Pei and Wei, 2019), but no new reports of USF FTLB have been emerging yet, which is likely attributed to the unavailability of long fluorescence lifetime USF CAs.

## Conclusions and Outlook

Nano-sized CAs of FLTB are one class of nanomedicines for disease diagnosis or monitoring. Small molecule-based CAs can fulfill the requirements of *in vitro* FLTB, such as cellular bioimaging, whereas they appear not applicable in tissue imaging due to the unavailability of NIR dyes. Even if a few NIR organic dyes have been developed and applied into bioimaging, scientists are still reluctant to use them in deep tissue bioimaging because of the poor imaging depth derived from large scattering light background. The use of nanomaterial-based CAs, such as QDs or UCNPs, improves the performance of *in vitro* FLTB in terms of a better quantum yield, the capability of simultaneous multiple-target imaging, and a better signal-to-background ratio (eliminating the autofluorescence), in comparison with that of small molecule-based CAs. Unfortunately, the goal of deep-tissue, high-resolution, and good contrast bioimaging appears far to reach for FTLB technique with nanomaterial-based CAs, as the optical bioimaging limit in deep tissue due to light scattering is inevitable. Thanks to their long fluorescence lifetimes, QDs- or UCNPs-CAs have been applied in tissue bioimaging with excellent contrast; however, the resolution in deep tissue is poor as a result of multiple scattering events of the emission light within tissue.

An intriguing breakthrough to this end was the hybrid bioimaging technique of ultrasound and fluorescence. Apart from the establishment of new ultrasound-modulated fluorescence imaging instrument, the development of CAs is a key element for the success of such hybrid bioimaging technique. Taking USF as an example, the CAs consist of a NIR dye-encapsulated nanocarrier. Intensity-base USF bioimaging have been explored, and results substantiate a proof-of-concept deep-tissue, high-resolution bioimaging. It is believed that USF-based FLTB will further improve the performance in deep tissue if a responsive lifetime-based CA is developed.

## Author Contributions

M-YW and QM oversaw the content, writing of the manuscript, and reviewed the finalized the manuscript. XL wrote the draft.

### Conflict of Interest

The authors declare that the research was conducted in the absence of any commercial or financial relationships that could be construed as a potential conflict of interest.
